# HIV testing and pre-exposure prophylaxis (PrEP) use, familiarity, and attitudes among gay and bisexual men in the United States: A national probability sample of three birth cohorts

**DOI:** 10.1371/journal.pone.0202806

**Published:** 2018-09-07

**Authors:** Phillip L. Hammack, Ilan H. Meyer, Evan A. Krueger, Marguerita Lightfoot, David M. Frost

**Affiliations:** 1 Department of Psychology, University of California, Santa Cruz, California, United States of America; 2 Williams Institute, UCLA School of Law, Los Angeles, California, United States of America; 3 Department of Medicine, University of California, San Francisco, California, United States of America; 4 Department of Social Science, University College, London, United Kingdom; Pontificia Universidade Catolica do Rio Grande do Sul, BRAZIL

## Abstract

This study examined HIV testing and use, familiarity, and attitudes toward pre-exposure prophylaxis (PrEP) among HIV-negative gay and bisexual men in the United States. A national probability sample (N = 470) of three age cohorts (18–25, 34–41, and 52–59 years) completed a survey between March, 2016 and March, 2017. Most men did not meet CDC recommendations for HIV testing, and 25.2% of men in the younger cohort had never tested. Only 4.1% used PrEP across cohorts. Visiting an LGBT clinic and searching for LGBT resources online were associated with PrEP use. Men in the middle cohort were more familiar with PrEP (79%) than men in the younger (52%) and older (57%) cohorts. Bisexual and non-urban men were less familiar with PrEP. Attitudes were positive among most men (68.4%) familiar with PrEP. Findings suggest that most men potentially at risk for HIV do not meet CDC guidelines for testing, and PrEP use continues to be minimal. Efforts to educate gay and bisexual men about HIV risk and prevention need to be reinvigorated and expanded to include non-gay-identified and non-urban men.

## Introduction

HIV continues to be a major public health concern, in particular for gay, bisexual, and other men who have sex with men in the United States, who account for 70% of new HIV infections annually[1]. Of these new infections, African American men account for the highest number (38%), followed by Latino (28%) and White (28%) men[2]. Over 60% of those who are undiagnosed are gay, bisexual, and other men who have sex with men[3], which is why the Centers for Disease Control and Prevention (CDC) recommends annual screening for this population. HIV testing is the entry point for accessing prevention and treatment.

The meaning of HIV and AIDS has shifted among gay, bisexual, and other men who have sex with men since the advent of major treatment and prevention advances[4–6]. Once considered a lethal diagnosis, HIV infection came to be seen as a chronic, manageable condition with the discovery of antiretroviral therapies in the mid-1990s[6]. This shift was accompanied by complacency about prior prevention methods such as condom use[7, 8] and called for new prevention approaches to reduce HIV transmission. The emergence of pre-exposure prophylaxis (PrEP) using Truvada as a highly effective tool in preventing transmission of HIV[9] signals a potential turning point in the public health and sexual culture of gay, bisexual, and other men who have sex with men. PrEP using Truvada was approved by the United States Food and Drug Administration (FDA) in 2012 and subsequently recommended by the World Health Organization (WHO) and the CDC for sexually active gay and bisexual who are HIV-negative and at substantial risk for HIV infection.

Although many men report an interest or willingness to use PrEP[10], the percentage of men taking Truvada for PrEP appears to be low, ranging from 0 to 12% in studies that rely on community samples in the USA[11–13]. The prevalence of PrEP use appears to be considerably lower than the CDC’s estimated 24.7% of men who have sex with men ages 18 to 59 who have indications for PrEP[14], though no research has examined PrEP use in a national probability sample to our knowledge. Research suggests that many men are ambivalent about PrEP[10] and may lack accurate knowledge about its effectiveness[15]. Given significant individual, structural, and social barriers to HIV testing[16, 17], many men with indications for PrEP may not be accessing information about its effectiveness.

Existing research on PrEP use has relied upon community samples of gay and bisexual men and has only minimally addressed the role that birth cohort may play in HIV testing or PrEP use, familiarity, or attitudes[18]. Because gay and bisexual men of distinct birth cohorts likely experienced the AIDS epidemic and sex education associated with it in unique ways[6], it is important to examine differences and similarities across birth cohort. For example, men over 50 experienced formidable trauma and loss with the AIDS epidemic, as they lost partners and entire social networks[5]. By contrast men in their 30s and 40s today were largely too young to have experienced these personal losses, but they experienced childhood and adolescence at the height of the epidemic and thus likely associated sex with other men with disease and death[6]. Men in this cohort also were socialized for strict condom use in a way that the prior generation had not. Men currently in early adulthood developed at a time when the meaning of HIV had transformed from a lethal illness to a chronic, manageable condition, and thus they likely view sex and sexual health through a different lens than both prior generations[6]. Studies with nonprobability samples of gay and bisexual men suggest that there is not an age difference for HIV testing[19]; however, younger men appear more willing to take PrEP than older men[20, 21]. No research to our knowledge has examined differences in use, familiarity, and attitudes across distinct birth cohorts.

Research with nonprobability samples of gay and bisexual men suggests possible differences in HIV testing and PrEP use based on race/ethnicity, though studies have somewhat conflicting findings. One study in HIV testing trends among young adults found a higher percentage of White males had tested than Black or Hispanic men[22]. Some studies suggest that Black men may be less aware[23–25] and less likely to use[26] PrEP than White men, which may be related to Black men’s distrust of health authorities such as the CDC[23, 27]. Other studies have suggested that White men may be more aware of PrEP[12] but may be less willing to use it[21, 23] than non-White men. The use of probability samples of racially diverse gay and bisexual men is needed to more closely examine differences in HIV testing and PrEP use, familiarity, and attitudes based on race or ethnicity. Our objective was to examine PrEP use, familiarity, and attitudes among a racially diverse US national probability sample.

## Materials and methods

### Sample and procedure

The current study examines data from male participants in the Generations Study. The Generations Study used a national probability sample of lesbians, gay men, and bisexuals in three age cohorts (18–25, 34–41, and 52–59 years old) recruited from all 50 US states and the District of Columbia. Cohort parameters were established based on identification of major historical events corresponding with critical points in the life course of sexual minorities[28]. Respondents were recruited in a 2-step process. A national probability sample was collected by Gallup using a dual-frame sampling procedure, with random-digit dialing of both landlines and cell phones. Respondents were interviewed using personal phone interviews that determined whether they identified as lesbian, gay, bisexual, or transgender (LGBT). Those responding affirmatively were then further assessed for eligibility. Respondents were eligible if they (a) identified as gay, lesbian, bisexual, queer, or same-gender loving, (b) were not transgender, (c) were Black, Latino, or White race/ethnicity, (d) were ages 18–25, 34–41, or 52–59 years old and (e) completed a sixth-grade education, and (f) answered the phone interview in English. (Transgender respondents were invited to participate in a concurrent study aimed at transgender participants). In the second step, eligible respondents who consented to be part of the survey self-administered a comprehensive online or mailed survey questionnaire. The results reported here were collected in the second-step survey.

In total, 366,644 participants were screened by Gallup between March, 2016 –March, 2017. Of them, 12,837 (3.5%) identified as LGB, 3,525 met eligibility criteria, 2,882 (82%) agreed to participate, and 1,345 (47%) completed the survey, for a total conditional participation rate of 38%. Participants responded to the survey by self-administering the study questionnaire either online via a link provided in an email or on paper via a mailed questionnaire returned in a pre-stamped, pre-addressed envelope. Participants read an information sheet prior to beginning the survey and consented by completing the questions and submitting it to the researchers. No signed consent forms were collected because of the self-administered nature of the data collection and because it was determined that a signed consent form, if it were collected, would impose an unnecessary risk to the respondents’ confidentiality. These procedures were approved by the Institutional Review Board of the University of California, Los Angeles.

The present analyses were limited to respondents who identified as male (N = 634, 47.1% of the sample). Of them, excluded from analyses were 10 (1.58%) who were of age that did not meet our cohort definitions; 67 (10.7%) men who were HIV-positive; and 87 (15.6%) men who had not had sex with another man in the five years prior to study. These exclusions resulted in a sample of 470 HIV-negative gay or bisexual men who are at some risk for HIV from sex with men.

### Measures

#### Outcome variables: HIV testing and PrEP use, familiarity, and attitudes

HIV testing was assessed with the question, “About how often do you get tested for HIV?” Response options were categorized according to having met CDC testing recommendations: Meets CDC testing recommendations (at least once a year), has tested but does not meet CDC testing recommendations (less frequently than once a year), or never tested.

PrEP use was measured using the question, “Are you currently taking Truvada as PrEP?” (yes/no).

PrEP familiarity was assessed with the question, “Truvada is a pill that HIV-negative people can take to prevent HIV infection. This is called PrEP (or Pre-Exposure Prophylaxis). How familiar are you with Truvada as PrEP?” Responses were dichotomized: “Not at all familiar” vs. “Familiar” (“somewhat familiar,” “very familiar”).

PrEP attitudes were assessed with the question, “Are you for or against HIV-negative people taking Truvada as PrEP to prevent the transmission of HIV?” Responses were dichotomized: “For it” (“I am for it”) vs. “not for it” (“I am against it,” “I have mixed feelings about it,” “I don’t have an opinion,” “I don’t know enough about it”).

#### Covariates

Demographic covariates included:

Age cohorts (18–25, 34–41, 52–59) were assigned based on date of birth.

Sexual identity was assessed with the question, “Which of the following best describes your current sexual orientation?” Response options were “straight/heterosexual,” “lesbian,” “gay,” “bisexual,” “queer,” “same-gender loving,” and “other.” Response options were collapsed into three categories: gay, bisexual, other.

Education was dichotomized (high school or less: “less than a high school diploma,” “high school graduate”; more than high school: “technical, trade, vocational, or business school or program after high school,” “some college,” “two-year associate degree,” “four-year bachelor’s degree from a college or university,” “some postgraduate or professional schooling after graduating college,” “postgraduate or professional schooling after graduating college”).

Race/ethnicity was assessed at the phone interview screening. Respondents were categorized into single-race categories as follows: Respondents who answered “yes” to the question, “Are you of Hispanic, Latino, or Spanish origin–such as Mexican, Puerto Rican, Cuban, or other Spanish origin?” were categorized as Latino/Hispanic, regardless of other races endorsed. Respondents who endorsed “Black or African American” were categorized as African American/Black, regardless of other races endorsed (with the exception of those categorized as Latino/Hispanic). Respondents were categorized as White if they endorsed White race including any additional race except for those endorsing African American/Black and those who were categorized as Latino/Hispanic.

Urbanicity scores were calculated using respondents’ residential zip codes according to the USDA Rural-Urban Commuting Area (RUCA) coding system (https://www.ers.usda.gov/data-products/rural-urban-commuting-area-codes.aspx). RUCA scores of 1–3 represented urban zip codes, while scores greater than 3 represented rural zip codes.

Psychosocial predictors included:

Relationship status: Respondents endorsing a current relationship (“are you currently in a relationship or feel a special commitment to someone?” [yes/no]) were asked “what is your current partner’s gender?” (women, non-transgender; men, non-transgender; transgender women/male-to-female [MTF]; transgender men/female-to-male [FTM]; non-binary/genderqueer). Those selecting “man, non-transgender” were categorized as such.

Perceived HIV risk HIV: Respondents were asked “how likely is it that you will become HIV-positive in your lifetime?” (very unlikely–very likely).

LGBT-specific health care: Respondents were asked, “In the past 5 years, how often have you been to an LGBT-specific clinic or provider for your healthcare?” (No: “never”; Yes: “sometimes,” “often”), and “during the past 12 months, have you looked for information online about certain health or medical issues” (LGBT-specific: “yes, an LGBT-specific website”; non-LGBT-specific: “no”, “yes, a general website”).

Out to health care providers: Respondents indicated if they were “out to all, most, some, or out to none of your healthcare providers.” We categorized as out those who said, “some,” “most,” “all,” and as not out those who said “none.”

LGBT community connectedness was assessed using a 7-item scale[29]. Scale items included, “you feel you’re a part of the LGBT community,” “you are proud of the LGBT community,” and “you feel a bond with the LGBT community” (4: agree strongly, 3: agree, 2: disagree, 1: disagree strongly). The mean value of all items present within the scale was recorded for each individual (range 1–4).

Internalized homophobia assessed respondents’ internalization of societal stigma against LGB people[30] using a 5-item scale. Scale items included “I wish I weren’t LGB” and “I feel that being LGB is a personal shortcoming for me” (1: strongly disagree, 2: somewhat disagree, 3: neither agree nor disagree, 4: somewhat agree, 5: strongly agree). The mean value of all items present within the scale was recorded for each individual (range 1–5).

Sexual identity centrality assessed the degree to which respondents’ LGB identities were central to their overall identities using a 5-item subscale from the 27-item Lesbian, Gay, and Bisexual Identity Scale (LGBIS)[31]. Scale items included “my sexual orientation is an insignificant part of who I am” and “being LGBT is a very important aspect of my life” (1: disagree strongly– 6: agree strongly). Items were reverse coded as necessary, and the scale was created as a mean score of all items present within the scale (range 1–6).

### Data analysis

Bivariate differences in HIV testing, PrEP use, familiarity, and attitudes were assessed by age cohort with design-based F tests using Stata 14. Multiple logistic regressions assessed differences in HIV testing and PrEP familiarity and attitudes, inclusive of all model covariates. We present full-model Odds Ratios and 95% confidence intervals. Due to the small number of people using PrEP and resultant large standard errors, we only conducted bivariate analyses for PrEP use. All analyses used survey weights to allow for generalization to the US population of HIV-negative gay and bisexual men within each of the age cohorts.

## Results

Table 1 shows prevalence and standard errors for HIV testing and PrEP use, familiarity, and attitudes in gay/bisexual HIV-negative men in the US.

**Table 1:** HIV testing, PrEP use, familiarity and PrEP, and attitudes toward PrEP among HIV-negative gay/bisexual men, U.S. population, by age cohort (N = 470).

	Full sample		Young cohort		Middle cohort		Old cohort		
	Count	Weighted Proportion (SE)	Count	Weighted Proportion (SE)	Count	Weighted Proportion (SE)	Count	Weighted Proportion (SE)	F
HIV Testing									
Never	67	16.94% (2.27)	45	25.15% (3.80)	8	7.65% (3.00)	14	7.84% (2.23)	10.49**
Yes, met CDC recommendation	212	46.51% (2.83)	88	45.27% (4.24)	63	58.53% (5.26)	61	35.99% (4.20)	4.71*
Using Truvada as PrEP	21	4.14% (1.13)	6	3.21% (1.62)	7	5.66% (2.55)	8	4.63% (1.73)	0.48
Familiar with PrEP	301	59.82% (2.81)	107	51.98% (4.28)	96	79.01% (4.56)	98	56.97% (4.44)	9.43**
Favorable attitude toward PrEP^†^	196	68.34% (3.19)	78	75.7% (4.68)	61	62.9% (6.02)	57	60.8% (5.45)	2.52

Note

*p < 0.05

**p < 0.01

^†^Among people who are familiar with PrEP only

### HIV testing

Of men in the young cohort, 25.2% (SE = 3.8) had never been tested for HIV, significantly more than men from the middle (7.7%, SE = 3.0) and older (7.8%, SE = 2.2) cohorts. Although this finding indicates that most men in all cohorts have tested for HIV at some time, in fact many men had not met CDC recommendation for HIV testing: only 45.3% (SE = 4.2) of the men in the young cohort, 58.5% (SE = 5.3) of men in the middle cohort, and 36.0% (SE = 4.2) of men in the older cohort had tested for HIV at least annually.

Table 2 shows results of multiple logistic regression predicting HIV testing behavior that meets CDC recommendations (adjusted full model ORs are reported). Men in the young cohort (AOR = 2.1, 95% CI = 1.1, 4.1) and middle cohort (AOR = 2.1, 95% CI = 1.1, 4.2) were more likely than men in the older cohort to meet CDC recommendation for HIV testing. Black respondents were more likely than White respondents to meet recommendations for HIV testing (AOR = 4.4, 95% CI = 1.6, 12.4), but White and Latino respondents did not differ. Also, as Table 2 indicates, gay, bisexual, and men who use other identity terms (e.g., queer) did not differ in testing behavior.

**Table 2:** Demographic and psychosocial correlates of HIV testing, PrEP use, familiarity and PrEP, and attitudes toward PrEP among HIV-negative gay/bisexual men, U.S. population, by age cohort (N = 470).

	Met CDC recommended HIV testing frequency		Familiar with Truvada as PrEP		Positive attitude about PrEP (Being "for PrEP")	
	N = 469		N = 469		N = 301	
	OR	95% CI	OR	95% CI	OR	95% CI
Demographic Predictors						
Cohort (ref = Older)						
*Middle*	2.15	(1.11, 4.16)*	3.37	(1.41, 8.06)**	2.14	(0.10, 5.27)
*Younger*	2.11	(1.10, 4.08)*	1.98	(0.97, 4.08)	3.03	(1.21, 7.58)*
Sexual identity (ref = Gay)						
*Bisexual*	1.05	(0.46, 2.37)	0.22	(0.092, 0.52)**	1.94	(0.39, 9.69)
*Other*	0.30	(0.11, 0.83)*	1.30	(0.35, 4.87)	0.35	(0.12, 0.97)*
Race/ethnicity (ref = White)						
*Black*	4.42	(1.58, 12.38)**	1.92	(0.65, 5.70)	0.40	(0.12, 1.34)
*Latino*	1.71	(0.84, 3.49)	1.06	(0.43, 2.64)	0.32	(0.14, 0.73)**
Education (ref = HS or less)						
*More than HS*	0.72	(0.38, 1.40)	2.94	(1.42, 6.08)**	0.41	(0.15, 1.11)
Urbanicity (ref = Urban)						
*Non-urban*	0.37	(0.13, 1.05)	0.26	(0.11, 0.63)**	0.52	(0.16, 1.67)
Psychosocial Predictors						
In a relationship with a man now (Yes)	0.90	(0.51, 1.58)	0.57	(0.30, 1.09)	0.67	(0.31, 1.45)
Tested for HIV (Yes)			4.24	(1.81, 9.94)**	2.71	(0.69, 10.55)
Likely to become positive (continuous)	1.91	(1.26, 2.88)**	1.02	(0.72, 1.45)	1.117	(0.70, 1.77)
LGBT clinic (Yes)	2.84	(1.36, 5.94)**	1.61	(0.66, 3.95)	0.52	(0.24, 1.12)
Looked online for LGBT info (Yes)	1.22	(0.49, 3.07)	2.57	(0.96, 6.86)	1.28	(0.60, 2.75)
Out to health care provider (ref = None)						
*Yes*, *some or more*	3.25	(1.30, 8.10)*	0.96	(0.44, 2.08)	2.34	(0.77, 7.13)
Community connectedness (continuous)	1.75	(0.98, 3.14)	1.21	(0.65, 2.25)	2.27	(1.22, 4.20)**
Internalized homophobia (continuous)	0.76	(0.53, 1.08)	0.97	(0.63, 1.51)	1.03	(0.65, 1.65)
Identity centrality (continuous)	0.78	(0.59, 1.02)	1.18	(0.86, 1.62)	1.09	(0.79, 1.50)

Note

* p < 0.05

** p < 0.01

Psychosocial predictors of meeting CDC recommendations for HIV testing included one’s assessment that he is at risk for HIV infection (AOR = 1.9, 95% CI = 1.3, 2.9), having visited an LGBT-specific clinic (AOR = 2.8, 95% CI = 1.4, 5.9), and being out to health care providers (AOR = 3.2, 95% CI = 1.3, 8.1).

### PrEP use

Overall, only 21 men, or 4.1% (SE = 1.1) of the total sample of HIV-negative sexually active gay and bisexual men reported taking Truvada as PrEP, which did not significantly vary by age cohort (design-based F = 0.48, p = 0.6). Bivariate logistic regressions revealed that bisexual men were less likely than gay men to use PrEP (OR = 0.1, 95% CI = 0.01, 0.9). Visiting an LGBT clinic and searching for information online on LGBT sources were associated with PrEP use (OR = 9.2, 95% CI = 2.7, 30.8; OR = 3.6, 95% CI = 1.1, 11.2, respectively).

### Familiarity with PrEP

Just above half the men in the younger and older cohorts (52.0%, SE = 4.3, and 57.0%, SE = 4.4, respectively) said they were familiar with the use of Truvada as PrEP, which is significantly fewer than men in the middle cohort, with 79.0% (SE = 4.6). Table 2 shows that demographic predictors of familiarity with PrEP include having more than high school education (AOR = 2.9, 95% CI = 1.4, 6.1), while having a bisexual identity (versus gay or other identities) and residing in a non-urban area were associated with reduced familiarity with PrEP (AOR = 0.2, 95% CI = 0.1, 0.5; AOR = 0.3, 95% CI = 0.1, 0.6, respectively). Psychosocial predictors of familiarity with PrEP include having tested for HIV (AOR = 4.2, 95% CI = 1.8, 9.9).

### Attitudes toward PrEP

Among men who were familiar with PrEP, most had a positive attitude toward it, which did not vary by age group with 68.4% (SE = 3.2) of the total sample reporting they were “for HIV-negative people taking Truvada as PrEP to prevent the transmission of HIV.” (However, in the fully controlled model, Table 2, more younger men had a favorable attitude toward PrEP than older men, AOR = 3.0, 95% CI = 1.2, 7.6).

Fewer men with other than gay or bisexual identity (e.g., “queer”) compared with gay men had a favorable attitude toward PrEP (AOR = 0.3, CI = 0.1, 0.98). Also, Latino men (compared with White men) were less likely to have a positive attitude towards PrEP (AOR = 0.3, 95% CI = 0.1, 0.7). Of the psychosocial variables, having a connection to the LGBT community was associated with positive attitudes toward PrEP (AOR = 2.3, 95% CI = 1.2, 4.2).

## Discussion

Despite recent public health efforts focused on educating gay, bisexual, and other men who have sex with men about HIV testing and promoting the availability of PrEP, our findings suggest that health educational efforts are not adequately reaching sizable and distinct groups of men at risk for HIV infection. In particular, our finding that 25% of young gay/bisexual men who are sexually active with a same-sex partner have never been tested for HIV is striking. It is, however, consistent with findings from a recent analysis that reported 29% of gay men aged 15–44 in the US have never been tested for HIV[32]. While men in the middle and older cohorts had higher levels of testing than the young cohort—which may be attributable to their older age and first-hand knowledge of the impact of HIV[6]—all of the cohorts had low levels of testing when considering CDC recommendations for HIV testing[33]. One exception is that Black respondents were more likely than White respondents to meet recommendations for HIV testing, which may be due to recent efforts to target Black men for testing, such as the CDC’s *Testing Makes Us Stronger* campaign. However, the overall low levels of testing suggest that there continue to be significant barriers to HIV testing among gay and bisexual men. Fear of knowing one’s status, low risk perception, anticipated stigma and discrimination, and conspiracy beliefs comprise individual barriers to testing uptake[16, 17]. These issues may be particularly salient for young men.

The number of PrEP users in the sample was extremely low (4.1%) but within the range of recent findings from nonprobability samples[13, 23, 34, 35]. Visiting an LGBT clinic and searching for information online on LGBT sources were significantly associated with PrEP use. The small number of respondents who reported PrEP use reduced the precision of our estimates, possibly leading to type-2 error (not detecting as significant associations that are in fact true).

Awareness of PrEP was also low, with only about 50% of men in the younger and older cohorts reporting familiarity with PrEP, compared to nearly 80% in the middle cohort. Heightened awareness in the middle cohort may be related to the unique role of HIV/AIDS in the course of these men’s lives, as they were saturated with information about AIDS during their childhood and adolescence in the 1980s and 1990s[6]. Given that increased likelihood of PrEP familiarity was associated with connections to LGBT-affiliated clinics and websites and living in urban locations, more efforts are needed to create knowledge of PrEP outside of urban LGBT communities.

Among those already familiar with PrEP, the majority expressed positive attitudes toward it. However, sizeable minorities within each cohort—one half to one third—did not express a positive attitude. More research is needed to understand factors contributing to attitudes toward PrEP in the broader population of gay, bisexual, and other men who have sex with men. It is noteworthy that our sample did not include men who identify as heterosexual or “mostly straight” and have sex with men. Men in our study identified as sexual minorities. Given the recent discovery that many men who have sex with men identify as straight or mostly straight[36, 37], future research needs to examine members of this unique population of men who are at high risk for HIV.

There are several limitations to the current study. We used a broad category of men as eligible for PrEP, defined here as men who have had sex with men in a 5-year period. CDC recommendations are much narrower and call for risk assessments that we were unable to make, including history of anal sex without condoms[38]. Given that the present study did not measure sexual risk behavior, it is not possible to correctly estimate the population of men who met current recommendations for PrEP use. Because of that, our estimate of 4% use is biased, with the actual proportion of eligible men who do not use PrEP probably lower. Finally, the way in which we asked the question about support for PrEP (“are you for or against HIV-negative people taking Truvada as PrEP to prevent the transmission of HIV?”) did not provide any information to respondents about PrEP effectiveness, adverse effects, or the meaning of “prevent.” Respondents unaware of PrEP may have interpreted the question in various ways. Future studies should further consider ways to assess support for PrEP in survey instruments.

### Public health implications

Our study is the first to examine HIV testing behavior, PrEP use, knowledge, and attitudes in a national probability sample of three distinct cohorts of HIV-negative gay and bisexual men. Our findings suggest the need to reinvigorate HIV prevention efforts to promote testing and awareness of PrEP, especially among younger gay and bisexual men and those who may not have access to LGBT communities and LGBT health providers. Although PrEP was approved over four years ago, there are many barriers to access to it, including affordability and lack of knowledge or access[15, 34, 35]. PrEP awareness may increase with access. In the meantime, public health efforts focused on promoting HIV testing and PrEP awareness need to be more inclusive of diverse populations of gay and bisexual men, especially those in rural areas who are less affiliated with the LGBT community, so that they and their health care providers can make informed decisions about PrEP use in managing risk for HIV.

## Supporting information

S1 File Data used for this paper are available at Generations W1_PLOS ONE_PrEP.xls. (XLS)
